# Repurposing fexofenadine as a promising candidate for diabetic kidney disease: randomized clinical trial

**DOI:** 10.1007/s11255-023-03804-w

**Published:** 2023-09-23

**Authors:** Basma Mahrous El-fatatry, Sahar Mohamed El-Haggar, Osama Mohamed Ibrahim, Khaled Hamed Shalaby

**Affiliations:** 1 Department of Clinical Pharmacy, Faculty of Pharmacy, Tanta University, Al-Guiesh Street, Tanta, 31527 Egypt; 2 Department of Clinical Pharmacy, Faculty of Pharmacy, Professor of Clinical Pharmacy, Tanta University, Al-Geish Street, Tanta, Egypt; 3 Department of Internal Medicine, Faculty of Medicine, Lecturer of Internal Medicine, Tanta University, Al-Geish Street, Tanta, Egypt

**Keywords:** Fexofenadine, Diabetic kidney disease, Albuminuria, Antihistamines

## Abstract

**Purpose:**

Diabetic kidney disease (DKD) is a devastating complication of diabetes mellitus. Inflammation and histamine are potentially involved in the disease progression. This study aimed to evaluate the role of fexofenadine in patients with DKD.

**Methods:**

From January 2020 to February 2022, out of 123 patients screened for eligibility, 61 patients completed the study. Patients were randomized into two groups, the fexofenadine group (n = 30): received ramipril plus fexofenadine, and the control group (n = 31): received ramipril only for six months. Changes in urinary albumin to creatinine ratio (UACR) and estimated glomerular filtration rate (eGFR) were considered primary outcomes. Measurements of urinary cyclophilin A, monocyte chemoattractant protein-1 (MCP-1), 8-hydroxy-2′ deoxyguanosine (8-OHdG), and podocalyxin (PCX) were considered secondary outcomes. The study was prospectively registered on clinicaltrial.gov on January 13, 2020, with identification code NCT04224428.

**Results:**

At the end of the study, fexofenadine reduced UACR by 16% (95% CI, − 23.4% to − 9.3%) versus a noticeable rise of 11% (95% CI, 4.1% to 17.8%) in UACR in the control group, (p < 0.001). No significant difference in eGFR was revealed between the two groups. However, the control group showed a significant decrease of − 3.5% (95% CI, − 6.6% to − 0.3%) in eGFR, compared to its baseline value. This reduction was not reported in the fexofenadine group. Fexofenadine use was associated with a significant decline in MCP-1, 8-OHdG, and PCX compared to baseline values.

**Conclusion:**

Fexofenadine is a possible promising adjuvant therapy in patients with DKD. Further large-scale trials are needed to confirm our preliminary results.

## Introduction

Diabetic kidney disease (DKD) is considered a leading cause of end-stage renal failure (ESRD). This microvascular complication occurs in about 40% of patients with type 2 diabetes mellitus [1]. Albuminuria is a typical sign of DKD and reduction of albuminuria is often associated with slowing of the disease progression [2]. Several pathologic structural and functional changes are seen in the diabetic kidney including metabolic alterations, oxidative stress, and activation of the local renin-angiotensin-aldosterone system (RAAS). Inhibitors of RAAS are often used to reduce albuminuria in patients with diabetes mellitus. Ramipril is one of the most widely prescribed anti-RAAS agents. Cianfrone et al. reported that the antiproteinuric response to ramipril is dose-dependent and increasing the dose above 5 mg/day led to a greater antiproteinuric effect of Ramipril [3]. Hyperkalemia and the inability to completely cure albuminuria are shortcomings of RAAS inhibitor use. Deterioration of albuminuria was reported in several studies despite using RAAS inhibitors [4–7]. Therefore, other treatment options are urgently needed to prevent the progression of DKD [6].

Inflammation is involved in the pathogenesis of DKD. Generation and circulation of advanced glycation end products lead to a release of reactive oxygen species and inflammatory mediators which result in glomerular hyperfiltration and albuminuria [2]. Moreover, a rise in the level of cell adhesion molecules, chemokines, and proinflammatory cytokines is observed in the renal tissues of diabetic patients and is correlated with albuminuria [8].

Histamine is potentially involved in the disease progression as the increase of degranulation status of mast cells in the diseased kidney leads to a release of histamine in the tubular interstitium which promotes renal inflammation and apoptosis [9]. Histamine H_1_-receptor antagonists are reported to have a direct anti-inflammatory effect and antagonize histamine-induced proinflammatory cytokine production [10, 11]. A preclinical trial reported the ability of antihistamines to improve renal function in diabetic rats and attenuate the elevated level of inflammatory cytokines like TNF-α and meliorated renal oxidative stress [11].

Fexofenadine is a selective histaminic H1 blocker with a favorable safety profile [12]. Like other H1-receptor antagonists, it is proven to have anti-inflammatory characteristics and suppress inflammatory cytokine release [13]. Based on the previous studies that reported the renoprotective effect of antihistaminic drugs and their effect in reducing protein excretion in DKD [11, 14], fexofenadine is expected to have a similar effect. Fexofenadine has the advantage that it doesn’t cause any degree of sedation even at high doses [15].

Given the complex nature of DKD and the predicted mechanism of action of fexofenadine, a number of biomarkers were chosen to be measured in the current study. Cyclophilin A is an abundant cellular protein with high expression in proximal tubular epithelial cells. It serves as an effective biomarker for the early detection of DKD [16]. Monocyte chemoattractant protein-1 (MCP-1) is a potent chemokine and remarkable inflammatory mediator that contributes to the pathogenesis of nearly all stages and all phenotypes of chronic and diabetic kidney disease [17]. Urinary 8-hydroxy-2’-deoxyguanosine (8-OHdG) is a sensitive biomarker for oxidative stress and has been reported to increase in patients with diabetes with micro- and macroalbuminuria compared to normoalbuminuric patients [18]. Podocalyxin (PCX) is a podocyte-specific protein that was reported to be an early marker for podocyte injury and diabetic nephropathy [19].

This study aimed to investigate the fexofenadine effect in reducing albuminuria in patients with diabetes mellitus. To the best of our knowledge, this is the first clinical study to evaluate the role of fexofenadine in DKD.

## Patients and methods

### Study design

This study was an open-labeled randomized controlled clinical study conducted in accordance with the ethical standards of Tanta University Research Ethical Committee, with the 1964 Helsinki declaration and its later amendments or comparable ethical standards. The study was prospectively registered on clinicaltrial.gov and its identification code is NCT04224428. The study record is available at https://classic.clinicaltrials.gov/ct2/show/NCT04224428.

Eligible patients were randomly assigned to the fexofenadine group and control groups. The two groups were randomized with a simple randomization method based on the hospital visit days. Patients were recruited from the hospital four days per week; 2 days for the fexofenadine group and 2 days for the control group. Patients in the fexofenadine group received ramipril plus fexofenadine 60 mg daily and patients in the control group received ramipril only for six months.

### Patients

The recruitment phase started in January 2020 at Internal Medicine Department, Tanta University Hospital, Tanta, Egypt. Inclusion criteria were age ≥ 18 years, confirmed diagnosis of Type 2 diabetes mellitus at least six months prior to screening, and stage 2 or 3 diabetic nephropathies (persistent micro- or macroalbuminuria with urinary albumin creatinine ratio (UACR) > 30 mg/g) despite treatment with ramipril 10 mg daily for at least 8 weeks prior to recruitment. Exclusion criteria were Type 1 diabetes mellitus, severe renal impairment (eGFR < 30 mL/min/1.73 m^2^), pregnancy or lactation, chronic heart failure, malignancy, inflammatory or autoimmune disease, and history of kidney disease other than diabetic nephropathy.

### Assessment

Personal data were obtained from each recruited patient at the screening visit including age, gender, height, weight, and body mass index (BMI). Urine samples were collected at baseline and after six months to assess UACR, cyclophilin A, MCP-1, 8-OHdG, and PCX using enzyme-linked immunosorbent assay (ELISA) kits. Analytes were performed using human cyclophilin A ELISA Kit with catalogue No. 201-12-0673, human monocyte chemoattractant protein-1 (MCP-1) ELISA Kit with catalogue No. 201-12-0125, human 8-hydroxy-2′ deoxyguanosine (8-OHdG) ELISA Kit with catalogue No. 201-12-1437, and human podocalyxin (PCX) ELISA Kit with catalogue No. 201-12-1835.The previous kits were purchased from Sunred Biological Technology Company, Shanghai, China. Blood samples were also collected at baseline and after six months to assess fasting blood glucose (FBG), glycosylated hemoglobin (Hemoglobin A_1_C), and serum creatinine using standard colorimetric methods.

The estimated glomerular filtration rate (eGFR) was calculated using the Chronic Kidney Disease Epidemiological Collaboration (CKD-EPI) equation as it is more accurate at higher levels of renal function and better to be used for clinical assessment of DKD [20]. Patients had regular visits every month for medication refills and to report any encountered side effects. Every month, medications administered by all patients were reviewed to exclude drugs that induce albuminuria or interact with fexofenadine.

The primary endpoint was the change in UACR, and eGFR after six months. Changes in other measured biomarkers were considered secondary outcomes.

### Statistical analysis

Statistical analysis was carried out using SPSS statistical package version 28.0, May 2021, IBM corporation software group, USA. A chi-square test was used to compare categorical clinical variables between groups. The Shapiro–Wilk test was applied to the measured parameters before running a parametric statistical analysis. The normality test revealed normally distributed data. Analysis of baseline characteristics and biomarkers were analyzed using an unpaired student t-test for parametric data. Whereas a student t-test was used to compare the percent change of variables of the two groups. Correlation analysis was done using Pearson correlation where correlation coefficients were interpreted as weak (< 0.4); moderate (0.4– < 0.7), or strong relationship (> 0.7) [23]. To evaluate the association between the measured biomarkers and UACR, a linear regression test was performed.

Considering that the primary objective of this trial was to compare UACR between the two groups, we calculated the minimum number of patients needed to detect the 20% change in UACR. The lower limit of 20% UACR reduction was chosen as a cut-off point representing clinical relevance which is not likely to be subjected to variance error. The assumed mean of the control group was 275 mg/g, and the expected mean difference between the control and treatment groups was 55 mg/g. We assumed 80% power, a two-sided type I error rate of 0.05, an allocation ratio (r = 1), and a standard deviation equal to 70 mg/g. After applying a 15% dropout rate, 30 participants were needed in each arm of the trial [21].

## Results

### Patient characteristics

**Fig. 1:**
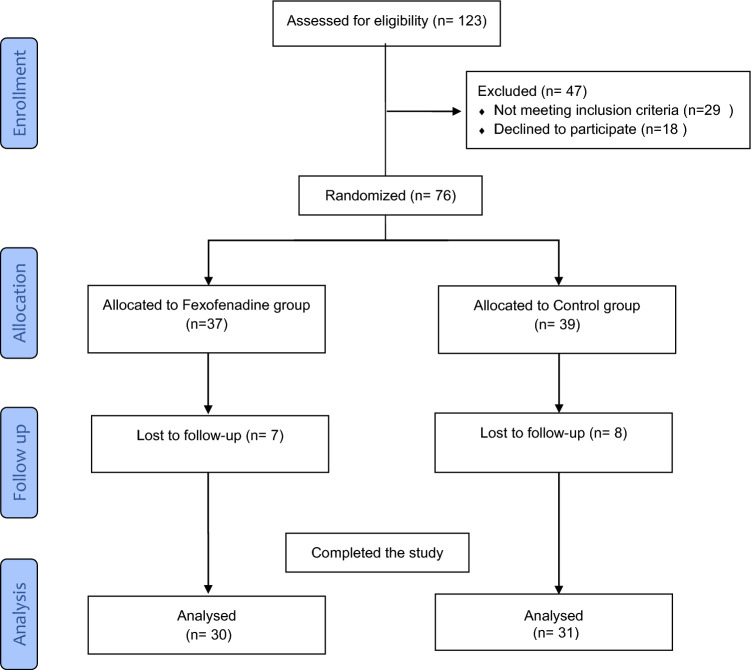
Flow chart of study participants

From January 2020 to February 2022, a total of 123 patients were assessed for eligibility. As shown in the flow diagram (Fig. 1), 76 patients were enrolled in the study and randomly assigned to the fexofenadine or the control group. Eight patients in the control group and seven patients in the fexofenadine group couldn’t be reached for the last outcome measure. Accordingly, 61 patients completed the study and were included in the per-protocol analysis. All patients were on ramipril 10 mg at least eight weeks before recruitment which continued throughout the entire study duration. All patients were on regular insulin and/or oral hypoglycemic agents to control their diabetes. No major changes have been made to their diabetic regimen throughout the study duration. Fexofenadine was well-tolerated, and no treatment-related side effect was reported. Baseline demographic and clinical characteristics were generally comparable in both groups (Table 1).

**Table 1:** Baseline patient characteristics

Characteristics	Fexofenadine group (n = 30)	Control group (n = 31)	P value
Gender, male, No. (%)	13 (43)	13 (41)	0.91
Age, mean ± SD, (Y)	55.4 ± 5.5	54 ± 5.8	0.36
Body mass index, mean ± SD, (kg/m^2^)	28.5 ± 3.4	29.2 ± 3.9	0.49
Hypertensive patients, No. (%)	15 (50%)	14 (45.2%)	0.7
Diabetes duration, mean ± SD, Years	9.17 ± 2	9.9 ± 3.3	0.31
Use of insulin, No. (%)	23 (76.7%)	26 (83.9%)	0.47
Fasting blood glucose, mean ± SD, (mg/dL)	180 ± 50	163 ± 26	0.1
Hemoglobin A1C, mean ± SD, (%)	9.6 ± 1.3	9.4 ± 1	0.45
Systolic blood pressure, mean ± SD, (mm Hg)	127 ± 14	131 ± 14	0.27
Diastolic blood pressure, mean ± SD, (mm Hg)	81 ± 7	87 ± 20	0.2

Values are expressed as mean ± standard deviation or number (percent)

### Primary outcomes

Fexofenadine significantly reduced UACR by 16% (95% CI, − 23.4% to − 9.3%) after six months while a significant rise of 11% (95% CI, 4.1% to 17.8%) in UACR was reported in the control group. Similarly, a statistically significant difference in mean values and percent change between the two groups were reported after six months of treatment (p < 0.001).

Mean values of eGFR were comparable in both groups and no significant difference was revealed between the two groups at the end of the study. However, the control group showed a significant decrease of − 3.5% (95% CI, − 6.6% to − 0.3%) in eGFR, compared to the baseline value. This significant reduction was not reported in the fexofenadine group (Table 2).

**Table 2:** Summary of the primary outcomes

	Fexofenadine group n = 30	Control group n = 31	P value 0 (between groups)
*UACR (mg/g)*			
Mean baseline	260 ± 53	248 ± 80	0.5
Mean after 6 months	215 ± 58	272 ± 85	0.004*
Percent change between the 2 points	− 16%	11%	< 0.001*
P value (within groups)	< 0.001*	0.004*	
*eGFR* (mL/min/1.73 m^2^)			
Mean baseline	82.5 ± 11.6	82.1 ± 18.5	0.92
Mean after 6 months	81.2 ± 12	78.3 ± 14	0.38
Percent change between the 2 points	− 1.5%	− 3.5%	0.31
P value (within groups)	0.144	0.016*	

Values are expressed as mean ± SD. *UACR* urinary albumin to creatinine ratio, *eGFR* estimated glomerular filtration rate

*Significant difference (P < 0.05)

### Secondary outcomes

Administration of fexofenadine for 6 months was associated with a significant decline in MCP-1, 8-OHdG, and PCX. On the other hand, the control group showed a significant increase in the levels of previously mentioned biomarkers during the same period compared to baseline values. (p < 0.05). Cyclophilin A didn’t differ markedly in the fexofenadine group after six months. In contrast to fexofenadine, a significant increase of 13.5% (95% CI, 6% to 21%) in cyclophilin A level was revealed in the control group after 6 months.

When comparing the percent change between the two groups, statistically significant differences were revealed in all measured biomarkers including cyclophilin A, MCP-1, 8-OHdG, and PCX in favor of the fexofenadine group (Table 3).

**Table 3:** Summary of the secondary outcomes

	Fexofenadine group n = 30	Control group n = 31	P value 0 (between groups)
*Cyclophilin A (ng/mL)*			
Mean baseline	25.4 ± 8	23.2 ± 6.3	0.25
Mean after 6 months	24.5 ± 7.6	26.2 ± 7.7	0.39
Percent change between the 2 points	− 1%	13.50%	0.01*
P value (within groups)	0.49	0.001*	
*MCP-1 (ng/L)*			
Mean baseline	325 ± 55	320 ± 78	0.75
Mean after 6 months	302 ± 56	343 ± 71	0.15
Percent change between the 2 points	− 6%	10%	< 0.001*
P value (within groups)	0.02*	< 0.001*	
*8-OHdG (ng/mL)*			
Mean baseline	34 ± 5.1	36 ± 7.2	0.16
Mean after 6 months	31 ± 4.5	40 ± 10	0.000*
Percent change between the 2 points	− 7%	14%	0.002*
P value (within groups)	0.002*	0.06	
*PCX (ng/mL)*			
Mean baseline	11.1 ± 4.7	9.5 ± 3.4	0.13
Mean after 6 months	8.5 ± 3.5	10.8 ± 4	0.02*
Percent change between the 2 points	− 20%	15%	< 0.001*
P value (within groups)	< 0.001*	0.001*	

Values are expressed as mean ± SD. *MCP-1* monocyte chemoattractant protein-1, *8-OHdG* 8-hydroxy-2’-deoxyguanosine, *PCX* podocalyxin

*Significant difference (P < 0.05)

A slight decrease in FBG and hemoglobin, A_1_C, − 0.05% (95% CI, − 0.09% to − 0.001%), and − 0.02% (95% CI, − 0.05% to − 0.01%) respectively were reported in the fexofenadine group. On the other hand, a slight increase in FBG, and hemoglobin A_1_C, 0.06% (95% CI, 0.006% to 0.11%), and 0.05% (95% CI, 0.02% to − 0.08%) respectively were reported in the control group. When comparing the percent change between the two groups, statistically significant differences were revealed (p = 0.003, p = 0.001 for FBG and hemoglobin A_1_C respectively).

Since hypertension may affect UACR, a sub-group analysis was performed. The baseline UACR of the 32 normotensive patients was not statistically different from that of the 29 hypertensive patients (253 ± 74 mg/g, vs 255 ± 60 mg/g respectively). Sub-group analysis showed that fexofenadine significantly reduced UACR in both normotensive patients and hypertensive patients (− 13.8% (95% CI, − 24% to − 4) vs − 18.8% (95% CI, − 30 to − 8) respectively) as shown in Table 4.

**Table 4:** Sub-group analysis of UACR based on hypertension

Hypertension	Fexofenadine group	Control group	P value between arms
Percent change in normotensive	(n = 15)	(n = 17)	0.003*
	− 13.8% ± 17	17.9% ± 23	
Percent change in hypertensive	(n = 15)	(n = 14)	< 0.001*
	− 18.8% ± 19	19.9% ± 12	

Values are expressed as mean ± SD. *UACR* urinary albumin to creatinine ratio

*Significant difference (P < 0.05)

The linear regression test showed non-significant results (p > 0.05). However, significant linear correlations were observed between UACR and cyclophilin A, MCP-1, 8-OHdG, and PCX biomarkers as shown in Table 5.

**Table 5:** Correlation between UACR and other measured biomarkers after 6 months

P value	Pearson correlation coefficient	Pearson correlation
Cyclophilin A	0.3	0.02*
MCP-1	0.4	0.004*
8-OHdG	0.4	0.003*
PCX	0.4	0.005*

*MCP-1* monocyte chemoattractant protein-1, *8-OHdG* 8-hydroxy-2'-deoxyguanosine, *PCX* podocalyxin, *UACR* urinary albumin to creatinine ratio

*Significant difference (P < 0.05)

## Discussion

To the best of our knowledge, the current study is the first randomized controlled study to identify fexofenadine’s role in reducing albuminuria in diabetic patients. Patients in the fexofenadine group showed a significant decline in albumin excretion and were protected from the significant increase in albuminuria and the significant decrease in eGFR that were reported in the control group.

Our finding is consistent with previous preclinical studies that reported the renoprotective effect of antihistaminic drugs and their effect in reducing protein excretion in DKD. Bilastine prevented the rise in UACR and levocetirizine reversed proteinuria in diabetic rats [11, 14]. The deterioration of albuminuria that occurred in the control group despite using ACEI is in accordance with several studies that reported an increase in albumin excretion and progression of DKD despite using RAAS blocking agents [4–7].

A suggested mechanism behind fexofenadine’s promising effect is protecting the kidney from inflammation which has a vital role in the initiation and extension of DKD. One convenient theory is that the number and degranulation status of mast cells increased in diabetic kidney suggesting that the histamine released increases the proinflammatory mediators and promotes renal tissue injury [9]. Fexofenadine’s effectiveness in DKD may be due to the reduction of kidney injury through direct anti-inflammatory or histamine-dependent anti-inflammatory effects.

In the current study, fexofenadine use was associated with a decrease in MCP-1 which is one of the key inflammatory cytokines involved in DKD. This finding is consistent with a previous preclinical study that reported the ability of levocetirizine to decrease the elevated renal level of inflammatory cytokines like tumor necrosis factor-α (TNF-α) in diabetic rats [11].

A significant positive correlation was revealed between UACR and MCP-1 at the end of this study. A similar significant association between albumin excretion and MCP-1 level in diabetic patients was reported in a recent clinical study [12]. This observation provides evidence of an association between albuminuria and inflammatory pathways within the diabetic kidney.

In addition to the anti-inflammatory effect, fexofenadine may affect DKD through its antioxidant effect. In the current study, fexofenadine not only prevented the increase of 8-OHdG that was seen in the control group but also resulted in a decrease in its level. In the same context, the antihistaminic drug, levocetirizine, reduced elevated levels of malondialdehyde and other oxidative stress markers in diabetic rats [11].

Intriguingly, the current study showed a significant positive correlation between UACR and 8-OHdG. Urinary level of 8-oxodG and 8-oxo-7,8-dihydroguanosine, which is a marker of oxidative stress, was also associated with an increased risk of albuminuria in patients with chronic kidney disease [22]. Therefore, our findings suggest an established relationship between oxidative stress markers and kidney function.

The significant reduction in PCX level in the fexofenadine group, and the significant correlation between UACR and PCX level suggest that fexofenadine’s anti-inflammatory, anti-oxidative effects protect against podocyte damage. Correspondingly, a previous preclinical study showed that bilastine reduced podocyte loss in the diabetic kidney and reported that H_1_ receptor antagonism could preserve podocyte integrity and filtration barrier [14]. Recent studies support an association between urinary PCX levels and albuminuria in diabetic patients [23, 24].

Moreover, the fexofenadine group showed slightly better glycemic control compared to the control group. In a recent previous study, the antihistaminic drug azelastine significantly reduced blood glucose, and hemoglobin A_1_C in diabetic rats [25]. Similarly, another preclinical study showed that levocetirizine in diabetic rats elicited a marked improvement in glycemic control [11]. The effect of H_1_ antagonism on diabetic glycemia may partially contribute to the fexofenadine-mediated effect on DKD in the current study.

This study has some potential limitations including a small sample size, short duration, and exclusion of patients with stage 4 and 5 chronic kidney disease.

## Conclusion

Fexofenadine may be a promising agent in reducing albuminuria in patients with type 2 diabetes receiving ACEI. Several mechanisms are suggested underlying fexofenadine efficacy including histamine-dependent and histamine-independent anti-inflammatory effects, antioxidant effects, decreasing podocyte injury, and improving glycemic control. Large-scale clinical trials with longer duration are needed to confirm our results.

## Data Availability

The datasets used during the current study are available from the corresponding author upon reasonable request.
